# New Insights into the Role of Exercise in Inhibiting mTOR Signaling in Triple-Negative Breast Cancer

**DOI:** 10.1155/2018/5896786

**Published:** 2018-09-30

**Authors:** Deborah Agostini, Valentina Natalucci, Giulia Baldelli, Mauro De Santi, Sabrina Donati Zeppa, Luciana Vallorani, Giosuè Annibalini, Francesco Lucertini, Ario Federici, Riccardo Izzo, Vilberto Stocchi, Elena Barbieri

**Affiliations:** ^1^Department of Biomolecular Sciences, University of Urbino Carlo Bo, Urbino, Italy; ^2^Interuniversity Institute of Myology (IIM), University of Urbino Carlo Bo, 61029 Urbino, PU, Italy

## Abstract

Triple-negative breast cancer (TNBC) does not express estrogen receptor, progesterone receptor, and human epidermal growth factor receptor 2 and is characterized by its aggressive nature, lack of targets for targeted therapies, and early peak of recurrence. Due to these specific characteristics, chemotherapy does not usually yield substantial improvements and new target therapies and alternative strategies are needed. The beneficial responses of TNBC survivors to regular exercise, including a reduction in the rate of tumor growth, are becoming increasingly apparent. Physiological adaptations to exercise occur in skeletal muscle but have an impact on the entire body through systemic control of energy homeostasis and metabolism, which in turn influence the TNBC tumor microenvironment. Gaining insights into the causal mechanisms of the therapeutic cancer control properties of regular exercise is important to improve the prescription and implementation of exercise and training in TNBC survivors. Here, we provide new evidence of the effects of exercise on TNBC prevention, control, and outcomes, based on the inhibition of the phosphatidylinositol-3-kinase (PI3K)/protein kinase B (PKB also known as Akt)/mammalian target of rapamycin (mTOR) (PI3K-Akt-mTOR) signaling. These findings have wide-ranging clinical implications for cancer treatment, including recurrence and case management.

## 1. Introduction

Breast cancer (BC) is one of the most common carcinomas and one of the main causes of cancer-related death worldwide [1]. Among the various subtypes, triple-negative BC (TNBC) accounts for approximately 20% of BC cases. The absence of estrogen and progesterone receptors and human epidermal receptor 2 (HER2) in malignant cells reduces treatment options and increases the risk of recurrence and death, especially in the first 3–5 years of follow-up after surgery [2]. Thus, TNBC exhibits a more aggressive clinical course than non-TNBC. Most TNBC cases are diagnosed in women under the age of 60, and in 20% of diagnosed cases, there is a mutation of the germinal BC (BRCA) gene [3–7]. In patients with metastatic TNBC, there are currently no available targeted therapies and chemotherapy is the only possible treatment option. In addition to the biological-molecular aspects associated with prognosis and BC development, a growing body of evidence highlights the impact of lifestyle on disease-related outcomes. Unhealthy lifestyles with low levels of physical activity (PA) result in overweight and obesity, which appear to have a negative impact on BC [8], increasing the risk of recurrence and death in all subtypes, including TNBC [9]. Conversely, proper diet, weight loss, and increased PA lead to more favourable outcomes in the short and long term [10, 11]. The mechanisms underlying the effects of exercise on breast carcinogenesis are not clear, but experimental evidence suggests that PA induces phosphatidylinositol-3-kinase (PI3K)/protein kinase B (PKB also known as Akt)/mammalian target of rapamycin (mTOR) (PI3K-Akt-mTOR) signaling inhibition and slows TNBC tumor cell growth [12–14]. Physiological adaptations to exercise occur primarily in skeletal muscle, but the effects of exercise and training also impact other tissues through systemic control of energy homeostasis and metabolism, thus influencing the TNBC tumor microenvironment and mTOR inhibition [15].

Given the scope of this review, we summarise recent discoveries related to the underlying biology of exercise-induced modulation of the mTOR pathway in TNBC, examining the benefits induced by different exercise and training protocols.

We also consider how exercise affects the level of microRNAs (miRNAs) linked to the mTOR pathway involved in TNBC initiation and progression [16, 17], and how nutrients can influence mTOR signaling.

Finally, we discuss how exercise induces beneficial adaptations and why it should be prescribed as a coadjuvant “medicine,” which has the potential to improve TNBC outcomes.

## 2. mTOR Signaling

### 2.1. mTOR Pathway and mTOR Activation in BC

mTOR is a serine-threonine kinase that interacts with several proteins to form two distinct complexes, mTORC1 and mTORC2, which show different sensitivities to rapamycin [18]. mTORC1 is acutely sensitive to rapamycin and responds to growth factors, stress, amino acids, and energy, promoting protein translation and synthesis, cell growth, mass, division, and survival. mTORC1 comprises mTOR, the regulatory associated protein of mTOR (Raptor), the G-protein *β*-subunit-like protein (G*β*L), also known as mLST8, DEP domain-containing mTOR-interacting protein (Deptor), proline-rich Akt substrate of 40 kDa (PRAS40), and Tti1/Tel2 complex. mTORC2 is insensitive to acute rapamycin treatment and contains mTOR, the rapamycin-insensitive companion of mTOR (Rictor), the mammalian stress-activated map kinase-interacting protein 1 (mSIN1), G*β*L, Deptor, protein observed with Rictor-1/2 (Protor 1/2), and Tti1/Tel2. Raptor and PRAS40 are unique to mTORC1, while Rictor, mSIN1, and Protor 1/2 are unique to mTORC2 [18].

The various components of mTORC1, which is the most widely studied complex, have several regulatory effects: Raptor, Tti1, and Tel2 are positive regulators, whereas PRAS40 and Deptor are negative regulators [19]. Several factors regulating mTORC1 activation converge in the tubular sclerosis complex (TSC), consisting of hamartin (TSC1), tuberin (TSC2), and TBC1 domain family member 7 (TBC1D7) [20]; the complex works via the Ras homolog enriched in brain (Rheb) GTPase, negatively regulating mTORC1 [21].

An upstream regulator of TSC is the PI3K/Akt pathway activated by growth factors such as insulin-like growth factor 1 (IGF-1) and insulin. PI3K phosphorylates phosphatidylinositol (3,4)-bis-phosphate (PIP2) lipid to phosphatidylinositol (3,4,5)-tris-phosphate (PIP3), which recruits phosphoinositide-dependent kinase-1 (PDK1) and Akt. Akt phosphorylates TSC2 and PRAS40 inactivating them and inducing, in turn, mTORC1 activation [22]. TSC2 can also be phosphorylated and inactivated by the activated Ras/extracellular signal-regulated kinase (ERK)/mitogen-activated protein kinase (MAPK) signaling pathway [19].

Another critical regulator of mTORC1 is the adenosine monophosphate-activated protein kinase (AMPK), which is activated when cellular energy level is low. AMP linking to AMPK allows its phosphorylation (while ATP availability prevents it) triggering repression of energy-consuming processes, also inhibiting mTOR, and enhancing energy-producing processes. AMPK phosphorylates TSC2 in different sites than Akt, activating rather than inactivating TSC2, and phosphorylates Raptor, thus achieving mTORC1 repression [23].

mTORC1 activation requires sufficient amino acid levels, though it is not clear how these levels are sensed. Amino acid regulation requires the formation of a Rag GTPase complex, which binds Raptor, in order to translocate mTORC1 to the lysosome allowing its association with Rheb, and thus its activation [24].

The activation of mTORC1 leads to several downstream effects, including protein synthesis promotion. Raptor binds to the eukaryotic translation initiation factor 4E- (eIF4E-) binding protein 1 (4E-BP1) and the ribosomal protein S6 kinase beta-1 (S6K1), recruiting them to the mTORC1 complex and allowing their phosphorylation [25, 26]. Hyperphosphorylation of 4E-BP1 by mTOR prevents the association of 4E-BP1 and eIF4E, allowing eIF4E to bind eIF4G to begin translation. Phosphorylation of S6Ks, including several S6K1 isoforms and S6K2, by mTOR promotes their activation and thus the phosphorylation of their targets involved in mRNA translation. S6K1 is also involved in negative feedback on mTORC1 and mTORC2 [27].

The mTORC1 complex and AMPK also regulate the autophagic process, a cellular mechanism through which cells eliminate damaged components associated with a wide range of diseases, including cancer. After glucose deprivation, AMPK associates with, and directly phosphorylates, the serine/threonine Unc-51-like autophagy activating kinase (ULK1), an upstream component of the autophagy mechanism. By contrast, when nutrients are plentiful, mTORC1 phosphorylates ULK1, preventing its association with and activation by AMPK, inhibiting autophagy [28].

Aberrant activation of the PI3K/Akt/mTOR pathway is often found in human cancers and promotes cell proliferation [29]. Activation has been shown in the lung, head, and neck and breast, gynaecologic, colorectal, and prostate cancers and glioblastoma multiforme [30] and also in B-lineage acute lymphoblastic leukemia [31]. PI3Ks are pivotal molecules in this pathway and possess eight isoforms grouped into class I, class II, and class III. Class I PI3Ks (PI3K*α*, *β*, *γ*, and *δ*), stimulated by Tyr kinases, G protein-coupled receptors, and Ras, are currently the focus of research in drug development. Mutation of the PIK3CA gene, which encodes the catalytic subunit *α* (p110*α*), one of the class I PI3K isoforms, is found in several cancers [32]. The signaling and biological roles of class II and III PI3Ks are not clear, and they have not been implicated in oncogenesis [32].

In TNBC, the activation of the PI3K/Akt/mTOR pathway is induced by an overexpression of upstream regulators (i.e., growth hormone receptors), mutations of the PIK3CA gene, and by decreased activity of the phosphatase and tensin homolog (PTEN) and of the proline-rich inositol polyphosphatase, which are downregulators of PI3K [33–35]. By contrast, activation of downstream effectors of PI3K (e.g., Akt and mTOR) and activation of downstream effectors of parallel pathways (MAPK and Ras) are rare events in TNBC [36]. Furthermore, other oncogenic pathways (i.e., FGFR, cMET, and RAF) regulated by P53 inactivation converge to activate the PI3K pathway [37].

Due to the frequent activation of the PI3K/Akt/mTOR pathway in human cancers, more than 50 inhibiting drugs are in development, and several clinical trials are ongoing [38]. The first established therapeutic anticancer agents targeting this pathway are everolimus and temsirolimus, which abrogate mTOR signaling, and have been approved by the U.S. Food and Drug Administration. Based on the results obtained with everolimus in pancreatic neuroendocrine tumors [39], and temsirolimus for advanced renal cell cancer [40], these agents are now approved for treatment of these diseases.

Therapies targeting other pathway members have been described. Monotherapy using pan-class I PI3K, which inhibits all class I PI3K isoforms, has effects at dose-limiting toxicity, leading to prolonged disease stabilization in some patients with advanced solid tumors (especially the lung) during phase I clinical trials [41]. Isoform-specific PI3K inhibitors have also been tested and have shown an antitumor activity in tumors such as p110*δ*- (isoform *δ*-) driven hematologic malignancies [42] or PIK3CA-mutant HR-positive BC [43]. Akt inhibitors and mTORC1/2 inhibitors aimed to suppress not only mTORC1, but also the feedback activation of Akt by mTORC2 [44], are currently being investigated in clinical studies [45]. The use of PI3K/Akt/mTOR pathway inhibitors is often associated with MAPK inhibitors, growth factor receptor inhibitors, and endocrine therapy. Furthermore, they might sensitize tumors to chemotherapy synergistically inducing apoptosis, as showed in sarcomas [46].

These promising strategies are now under investigation for the treatment of several tumors, including nonsmall cell lung cancer [47], colorectal cancer [48], nonmedullary thyroid carcinoma [49], and B-lineage acute lymphoblastic leukemia [31].

Although these strategies have been shown to be effective, there is great variability in the duration and quality of their benefits and the long-term side effects for patients. Thus, the identification of protein and/or genetic biomarkers to recognize subjects that will benefit the most from these therapeutic strategies is essential [50]. In TNBC, the development of PI3K/Akt/mTOR-targeted therapies, taking into account the inhibitors of this pathway alone or in combination with other strategies, will provide new tools to control disease progression and improve outcomes [51]. In a recent phase 2 clinical trial, the efficacy of ipatasertib (an Akt inhibitor) in association with paclitaxel (an antineoplastic agent used in TNBC treatment) was shown [52].

### 2.2. MicroRNAs and mTOR Signaling in BC

Several studies highlight the role of circulating microRNAs (miRNAs), in different tumors, including BC and the TNBC subtype [16]. In particular, recent evidence has shown that miR10a is downregulated in triple-negative BC cells [53]. Furthermore, overexpression of miR-10a decreases the proliferation and migration of TNBC cell lines via PI3K/Akt/mTOR signaling and through the mitochondrial apoptotic pathway [53]. Recently, Phua et al. [54] demonstrated that miR-184 is also downregulated in TNBC patients and that miR-184 overexpression in TNBC cells leads to a reduced expression of mTOR. The decreased cancer cell proliferation, due to mTOR reduction, has been confirmed *in vivo*: mice injected with mir-184-transfected MDA-MB-231 cells showed a delayed primary tumor formation and reduced metastatic burden. Emerging evidence points to epigenetic silencing by hypermethylation as a possible mechanism through which these tumor suppressor/growth inhibitor miRNAs are downregulated in TNBC [55]. In metastatic breast tumors, miR-184 has been found to be hypermethylated compared to the methylation status of miR-184 in normal breast tissue, suggesting a selective pressure in silencing this miRNA during the metastatic process [54].

Upregulation of miR-21 was detected in TNBC tissues and in MDA-MB-468 cells by Fang et al. [56]. Inhibition of this miRNA resulted in decreased proliferation, viability, and invasiveness of TNBC cells and enhanced apoptosis. Experiments to identify miR-21 targets have shown that PTEN is downregulated, suggesting an activation of mTOR and the oncogenic properties of miR-21 in TNBC, with increased proliferation and invasion by TNBC cells. Another miRNA that has been found to be upregulated in TNBC tissues in comparison to non-TNBC or adjacent tissues is miR-146a. Indeed, it has been reported to be significantly related to tumor size and histological stage: patients with elevated miR-146a expression have lower survival rates and worse prognoses than low-expression individuals [57]. In addition, miR-146a has been shown to bind the 3′-UTR region of BRCA1, inhibiting its expression; the BRCA1 protein is absent or present at very low levels in about one-third of sporadic BCs [58]. Evidence suggests that downregulation of BRCA1 expression leads to Akt/mTOR oncogenic pathway activation [59]. Hence, strategies that could modify the deregulated status of these miRNAs in TNBC could have a pivotal role in inhibiting the Akt/mTOR pathway and could affect TNBC initiation and progression. It is not yet known how these miRNAs might be modulated by exercise and whether they can be associated positively or negatively with TNBC progression, for which there are no reliable prognostic factors.

### 2.3. Autophagy and mTOR Signaling

Autophagy is the cellular mechanism responsible for the degradation of cytoplasmic components. It is through this mechanism that cells maintain cellular homeostasis by eliminating damaged proteins and organelles and by providing substrates for energy generation and biosynthesis under stress conditions. The mTOR complex is a major negative regulator of autophagy. It suppresses autophagy in response to nutrients, growth factors, and hormone availability, promoting protein synthesis, cell division, and metabolism. The mTOR signaling pathway is frequently activated in tumor cells, resulting in the activation of its growth-promoting functions and the inhibition of autophagy [60]. In cancer, the cytoprotective role of autophagy could prevent tumorigenic transformation by inhibiting chronic tissue damage. By contrast, once cancer occurs, cancerous cells could utilize autophagy to enhance fitness and survive in the hostile tumor microenvironment, providing energy via substrate degradation. Autophagy could therefore be tumor suppressive (for example, via elimination of damaged cellular components), as well as tumor promoting in established cancers [61]. In addition, autophagy has recently been shown to play a role in necroptosis, and, together with apoptosis, autophagy also regulates other death pathways, including immunogenic cell death, entosis, and pyroptosis [62]. It has been demonstrated that suppression of autophagy in epidermal growth factor receptor- (EGFR-) driven nonsmall cell lung adenocarcinoma xenografts promotes cell proliferation, tumor growth, and dedifferentiation, as well as resistance to EGFR tyrosine kinase inhibitor therapy [63]. Moreover, autophagy suppresses early oncogenesis in lung adenocarcinoma through effects on regulatory T cells [64], and autophagy genes are often required for the cytotoxic effects of chemotherapy [65]. In view of the complex- and context-dependent role of autophagy in cancer progression and response to therapy, it could be hypothesized that the inhibition of the mTOR pathway and the consequent induction of autophagy may be useful in certain cancers through autophagy-dependent antitumor immunity, autophagy-dependent cytotoxic effects, or other tumor-suppressor effects [66]. In addition to its effects on skeletal muscle, exercise has also been found to induce autophagy in the liver, pancreas, adipose tissue, and cerebral cortex in transgenic mouse models [67, 68]. Whether exercise-induced stress activates autophagy in healthy cells (or cells primed for malignant transformation), or cancer cells themselves, and whether such effects inhibit or potentiate tumorigenesis, is not known and needs further investigation [15].

## 3. Evidence of mTOR Modulation by Exercise in TNBC

### 3.1. mTOR and Exercise

PA reduces mortality for all diseases, including tumors [69], reducing the incidence of primary development and ameliorating the prognosis [15]. Hence, it should be prescribed like a medication indicating the correct typology, dose, and timing, i.e., the type, intensity, duration, and frequency of exercise as described in Exercise Prescription in BC Survivors. Physiological adaptations to exercise occur not only in skeletal muscle but also systemically in other metabolically active tissues involved in the exercise response (such as the bone, heart, adipose, endothelium tissue, and brain) profoundly altering the systemic milieu, in turn influencing the tumor microenvironment and cancer hallmarks [15]. In order to understand the effect of PA on mTOR and BC, muscular, systemic, and microenvironment effects should be considered.

#### 3.1.1. Aerobic Exercise and Muscular Effects

In skeletal muscle, aerobic exercise activates several adaptive pathways, including protein kinases, transcription, and coregulatory factors that, by gene expression modification, increase mitochondrial biogenesis and stimulate metabolic reprogramming [70]. Exercise induces a depletion of nutrients, energetic substrates, and nicotinamide adenine dinucleotide (NAD)H that elevate the ratios of AMP : ATP and NAD+ : NADH, directly activating AMPK and other metabolic sensors, including NAD-dependent protein deacetylase sirtuin 1 (SIRT1) and kinases, such as ERK1/2, p38 MAPK, and Jun N-terminal kinase (JNK) [71]. These energy sensors trigger the transcriptional regulator peroxisome proliferator-activated receptor-*γ* coactivator 1*α* (PGC1*α*), which regulates the expression of mitochondrial biogenesis, increase the expression of mitochondrial transcription factor A (TFAM), which, once transferred to the mitochondria, controls transcription of mitochondrial DNA [71]. Moreover, aerobic exercise, through PGC1*α* phosphorylation, influences other transcription factors, including peroxisome proliferator-activated receptor-*γ* (PPAR*γ*), an important regulator of fatty acid oxidation and estrogen-related receptor-*α* (ERR*α*) and ERR*γ*, which directly regulate mitochondrial energy metabolism by oxidative phosphorylation, fatty acid oxidation, and the tricarboxylic acid (TCA) cycle [72, 73]. In this regard, the reactive oxygen species (ROS) and reactive nitrogen species produced by exercise also directly or indirectly regulate contraction-induced mitochondrial biogenesis [74] and skeletal muscle metabolic reprogramming via AMPK and PGC-1*α* [75]. AMPK-mediated cell survival requires inhibition of mTOR. Therefore, AMPK and mTOR play antagonistic roles in cells and inhibition of mTOR is essential for AMPK-mediated metabolic homeostasis [76].

#### 3.1.2. Resistance Exercise and Muscular Effects

In skeletal muscle, resistance exercise causes an increase in muscle size and strength via mTOR activation. In canonical growth factor signaling, mTOR is activated by PI3K/Akt, through IGF-1 and insulin signaling, but a considerable body of evidence suggests that mTORC1 is also likely activated by a growth factor-independent movement of proteins to and from the lysosome, via resistance exercise-induced phosphorylation of TSC2 [77]. Cellular trafficking of mTOR and its association with positive regulators that occur in human skeletal muscle leading to protein synthesis after resistance exercise, in fed condition, were recently confirmed by Song and colleagues [78].

#### 3.1.3. Systemic and Microenvironment Effects of Exercise

**Figure 1:**
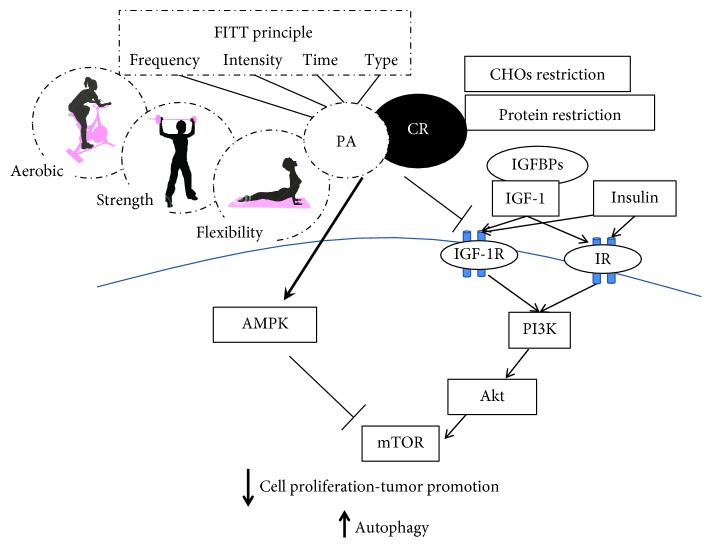
In this figure, we consider potential mechanisms regulated by physical activity and caloric restriction in inhibiting the mTOR pathway. Both refer to energy availability inhibiting carcinogenesis by suppressing the activation of the mTOR signaling network in this subtype of mammary carcinoma. The mTOR inhibition is mediated through the effects of vigorous PA or long-term exercise on systemic response such as concentrations of the circulating growth factors and hormones (i.e., IGF-1 and insulin) that regulate the mTOR network. The network is controlled through the PI3K/Akt signaling pathway, the glycaemia and glutamine levels, inducing apoptosis and reversing malignancy-associated metabolic programming. Moreover, the control of energy availability by both exercise and CR induces a mitohormetic response that accounts for a physiological cellular stress adaptation through AMPK activation inducing mTOR inhibition. In this context, exercise should be considered in terms of its four components: frequency, intensity, time, and type; however, dose-dependent effects of each component on cancer protection via mTOR inhibition have not yet been clarified. Most data indicate that vigorous PA, either long-term or in adulthood, may reduce a woman's risk of mammarian cancer, especially TNBC relapse. The inhibition of the mTOR complex and its cell growth-promoting functions leads to a reduction of cell proliferation, control of cancer progression, and consequent autophagy induction probably involved in tumorigenesis prevention. Thus, we hypothesized that the exercise-induced inhibition of the mTOR pathway may be useful in the control of cancer progression, including TNBC. PA: physical activity; CR: caloric restriction; CHOs: carbohydrates; mTOR: mammalian target of rapamycin; IGF-1: insulin-like growth factor 1; IGF-1R: insulin-like growth factor receptor 1; IR: insulin receptor; IGFBPs: insulin-like growth factor binding proteins; PI3K: phosphatidylinositol-3-kinase; AMPK: adenosine monophosphate-activated protein kinase; TNBC: triple-negative breast cancer. FITT-VP principle, which reflects the frequency (*F*), intensity (*I*), time (*T*), and type (*T*) of exercise, and its volume (*V*) and progression (*P*) over time, in an individualized exercise training program.

Exercise stimulates the release of molecular signals such as muscle-derived regulatory RNAs, metabolites, and myokines with autocrine, paracrine effect on energetic substrate oxidation, hypertrophy, angiogenesis, inflammation, and regulation of the extracellular matrix. To better evaluate the systemic response to PA, a distinction must be drawn between long term (training) and acute exercise. Training induces a reduction of basal concentration of circulatory sex hormones and lowers adiposity, both recognized risk factors [79], while acute exercise causes a sharp increase in circulating hormones, cytokines, and immune cells [80–82]. Both the systemic adaptations to training and the strong response to acute exercise support plausible mechanisms that inhibit carcinogenesis by suppressing the activation of mTOR signaling network. Hence, exercise may improve BC outcomes [14] (Figure 1). Moreover, both long-term training and a single bout of exercise control energy availability and induce a hormetic response that accounts for the physiological cellular stress adaptation [83, 84].

Hormesis is a process whereby exposure to a low dose of a potential stress favours adaptive changes in the cell that enables it to better tolerate subsequent stress [85, 86]. This type of stress is often related to reactive oxygen species (ROS) originating from the mitochondrial respiratory chain [87]. The accumulation of transient low doses of ROS through exercise influences signaling from the mitochondrial compartment to the cell [88]. Remarkably, this coordinated response to mild mitochondrial stress appears to induce mitochondrial metabolism, increase stress resistance, stimulate various long-lasting cytoprotective pathways, and favour the establishment of an oxidant-resistant phenotype, hence preventing oxidative damage and chronic diseases. Accordingly, low levels of ROS elicit positive effects on physiological cellular and systemic responses and ultimately increase lifespan [83, 88–93]. The hormetic nature of exercise, which produces low levels of ROS, emerges as a key feature for cancer control. Indeed, in the tumor microenvironment, the activation of exercise-induced hormesis of the AMPK-p38-PGC1-*α* axis supports oxidative metabolism maintaining the cellular ATP pool and conserving cellular energy and viability during the metabolic stress condition: AMPK regulates metabolism and energy homeostasis [94, 95]. Exercise-induced mitochondrial biogenesis improves mitochondrial function in addition to the upregulation of antioxidant defenses that function as back regulators of intracellular ROS levels, and leads to improved redox homeostasis [96, 97] as well as significantly improved insulin sensitivity. By contrast, high levels of ROS cause functional oxidative damage to proteins, lipids, nucleic acids, and cell components, induce a significant increase in intracellular Ca^2+^, and promote signaling cascades for apoptosis or autophagy via NF-*κ*B or forkhead box sub group O (FoxO) pathways. High ROS levels are therefore reputed to act as etiological, or at least exacerbating factors in chronic/aging-related diseases.

The typical hormetic response modulated by exercise involves kinases, deacetylases, and transcription factors; many of which have also been shown to be involved in the carcinogenic process [86]. The most studied are sirtuins (SIRT), which are histone deacetylases, and the FoxO family of transcription factors. The pathways in which NF-kappaB and the Nrf-2/ARE are components are also involved in hormetic responses and implicated in carcinogenesis and are modulated by exercise [86].

FoxO transcription factors play a critical role in cell cycle control and cellular stress responses. FoxOs are known to be regulated by the insulin signaling pathway; however, recently, the research group of Burnet demonstrated that AMPK phosphorylates 6 specific residues on FoxO and opposes the phosphorylation of other FoxO sites by Akt [98]. Phosphorylation of FoxO by AMPK affects the conformation of the protein in such a way that sirtuin-mediated deacetylation is also modified [99]. The dependence of sirtuins on nicotinamide adenine dinucleotide (NAD(+)) links their activity to cellular metabolic status. Emerging evidence indicates that deacetylation of FoxO by SIRT1 favours expression of cell survival/stress resistance and the downregulation of proapoptotic genes [85, 100, 101]. Sirtuins therefore protect against cancer development as they regulate the cellular stress responses and ensure that damaged DNA is not propagated and that mutations do not accumulate [99]. However, how FoxO activation is influenced by exercise remains unclear. In addition, cytokines such as those that we and others have found to be regulated by exercise and training [14, 102–104] have been reported to have direct and indirect effects on cellular stress responses modulated by acetylation/deacetylation reactions, and these effects can be further modified by cortical steroids, which exercise dramatically induces [105].

Similarly, various chemical mimetics of PA and caloric restriction (CR) such as AICAR, PPAR*δ* agonist, resveratrol, and metformin can trigger a beneficial response by activation of key regulators of stress tolerance at the level of transcription, posttranscriptional modifications, and regulation of energy metabolism [92, 106]. Cross talk between major CR hormesis-induced pathways, especially AMPK/PPAR and antioxidant systems, IGF-1, and homeostatic energy balance, reveals the correlation between CR and exercise mimetics [107].

Likewise, depending on the exercise, the level/persistence could induce an adaptive response that might turn the same process from “physiologic” into “pathologic,” as in the case of inflammation. Careful titration of ROS levels within specific tumor microenvironments may lie at the crossroads between the prevention, protection, and/or initiation and progression of disease, in particular, as regards the induction of mitochondrial functionality, cellular homeostasis, and more generally, cellular metabolic health.

Considering the type of exercise, both aerobic and resistance training increase glucose uptake in skeletal muscle via insulin-independent mechanisms, with a subsequent decrease in circulating levels of insulin, IGF-1, and glucose [108]. In a model of mammary carcinogenesis, PA caused a delay in carcinogenesis with a concomitant activation of AMPK and reduction in Akt and mTOR activation and reduction in insulin and IGF-1 in circulation [12]. Reduction of insulin levels is an important aspect given that hyperinsulinemia and insulin resistance are commonly observed in obesity with adipokine alterations, conditions associated with increased risk of BC and poor prognosis [8]. Insulin resistance is a condition in which the target tissues of insulin such as skeletal muscle, adipose tissue, and liver show a reduction in their response to physiological concentrations of the insulin hormone. As a consequence, the pancreatic *β*-cells produce more of the hormone to compensate for the defective response of target tissues, thus leading to hyperinsulinemia. BC cells express high levels of the insulin receptor (IR), and increased circulating insulin is associated with BC recurrence and death [109]. In contrast, PA has a fundamental role in reducing muscle insulin resistance and normalizing circulating insulin levels. Regular exercise in both healthy and oncological conditions ameliorates glycemic control including glycated hemoglobin (HbA1c) and insulin sensitivity in a “dose”-dependent manner according to duration and intensity [110, 111]. Skeletal muscle in virtue of its mass and high rate of insulin and exercise-stimulated glucose transport, represents the most important tissue in glucose uptake. Exercise per se increases trafficking of glucose transporter 4 (GLUT4) to the plasma membrane through insulin-independent mechanisms [112]. Under normal physiological conditions, in skeletal muscle, insulin actions are mediated by the IR-catalyzed phosphorylation of the IR substrates 1 and 2 (IRS1 and IRS2). The tyrosine-phosphorylated IRS proteins then interact with and activate PI3K, a critical player in insulin signaling, particularly with regard to glucose homeostasis. Activation of PI3K generates PIP3 that induces membrane translocation of the serine/threonine kinase Akt. PIP3 activation of PDK1 and the Rictor/mTOR complex 2 leads to phosphorylation and subsequent activation of Akt [113]. Akt phosphorylates TBC1D4 (also known as Akt substrate of 160 kDa, AS160) and TBC1D1 promoting the translocation of GLUT4 vesicles from intracellular compartments to membrane for glucose uptake [114].

Although recent findings help to better understand the effect of exercise on glycemic control, the specific exercise-induced signaling mechanisms leading to the acute and long-term adaptations favouring enhanced glycemic control are less clear [112, 115].

Endurance and, to a lesser extent, resistance exercise represent a significant metabolic stress, activating AMPK and thus inhibiting mTOR also in nonmuscular tissue such as liver, fat, and tumor tissues. In order to better evaluate the impact of exercise on mTOR in the BC microenvironment, not only AMPK, but also other circulating factors, should be considered. IGF-1, as well as insulin, activates the MAPK pathway and the PI3K pathway, which are both involved in cancer development and progression. The importance of IGF-1 axis in the development and progression of BC has been clearly shown [116]. The overexpression of IGF-1R in BC has been reported and related to poorer survival rates [117].

The IGF signaling system is composed by IGF-1 and IGF-2, insulin-like growth factor binding proteins (IGFBPs), a family of binding proteins regulating IGF half-lives and available in circulation and extracellular fluids, IGF receptors, and insulin receptors. Furthermore, we recently evaluated the complexity of the IGF-1 gene [118] and the biological activity of IGF-1 isoforms in BC cell lines [119] showing that the IGF-1 isoforms induced cell proliferation via IGF1R phosphorylation. Some studies have reported conflicting results regarding the regulation of IGF-1. Such studies report an increase, no difference or a decrease in circulating IGF-1 levels associated with PA [120–123]. These results are not surprising because the IGF-1 levels are influenced by several clinical factors such as gender, age, body mass index (BMI), sex steroid concentrations, nutrition, stress, level of PA, and intervening illness. Thus, exercise prescription should take into consideration most of these variables.

Another process through which exercise might regulate tumor metabolism is the autophagic machinery [15], as described in Autophagy and mTOR Signaling.

It is clear that exercise can ameliorate the BC microenvironment and can be very important in reducing BC risk and tumor burden when canonical radiochemotherapies or chemical mTOR inhibitors are not working, as in TNBC. Exercise workouts for these subjects will be explained in Exercise Prescription in BC Survivors. Ex vivo experimental data, using TNBC cell lines stimulated with sera collected before and after a single aerobic exercise bout (pre- or postexercise serum/a), are described in Experimental Evidence of mTOR Inhibition.

### 3.2. Experimental Evidence of mTOR Inhibition

As regards the mechanisms involved in the exercise-induced reduction of TNBC risk and tumorigenesis, few data are available. Ex vivo experiments, working with TNBC cells stimulated with sera collected before and after a single aerobic exercise bout (pre- or postexercise serum/a), are a good starting point to understand how exercise could affect the progression and recrudescence of TNBC. The research group of Dethlefsen has demonstrated that incubation of MCF-7 estrogen-responsive BC cells and MDA-MB-231 TNBC cells treated with postexercise serum, from both healthy volunteers [124] and operated cancer patients [14, 124], resulted in a reduction of BC cell viability in comparison with BC cells incubated with preexercise sera. In particular, it has been demonstrated that MCF-7 and MDA-MB-231 stimulation with sera leads to a viability reduction of 11% in MCF-7 cells and 9% in MDA-MB-231 cells in the case of supplementation with postexercise serum from operated cancer patients receiving adjuvant chemotherapy compared to preexercise serum [124]. Furthermore, the viability of both BC cell lines supplemented with sera from healthy women was also significantly reduced by the exercise-conditioned sera, resulting in a 10% and 19% reduction in MCF-7 viability and a 14% and 13% reduction in MDA-MB-231 viability by 1 h and 2 h postexercise sera, respectively. The reduced viability of MDA-MB-231 supplemented with 5% of healthy women 2-hour postexercise serum has also been confirmed by a pilot study that we performed working with culture medium with a physiological concentration of glucose (80mg/dl), resulting in a statistically significant reduction in cell proliferation of about 10% compared to cells supplemented with preexercise human serum [103]. Promising data on the tumorigenic potential of cancer cells in mice are also available. As reported by Dethlefsen et al. in 2017 [124], different outcomes in incidence and growth of tumors were detected inoculating NMRI-Foxn1nu mice with MCF-7 or MDA-MB-231 BC cells preincubated for 48 hours with pre or postexercise sera from healthy volunteers. In particular, only 45% of the mice inoculated with MCF-7 supplemented with postexercise human serum formed tumors compared with 90% of mice inoculated with MCF-7 preincubated with at rest sera, and the volume of tumors was reduced by 76%. Moreover, tumor incidence in mice inoculated with MDA-MB-231 cells preincubated with postexercise sera tended to be lower than it was in mice inoculated with MDA-MB-231 cells preincubated with rest sera, but no difference in tumor volume was observed between the two groups. These results show that exercise-stimulated changes suppress BC cell proliferation and reduce the tumorigenic potential of BC cells, also in the case of TNBC cells. Another important aspect to be considered is the fact that PA has been reported to lead to an increased level of the catecholamines epinephrine (EPI) and norepinephrine (NE) [82]; this result has also been confirmed in BC survivors two hours after a single exercise session [124]. Moreover, by blocking the *β*-adrenergic signaling pathway in BC cells, the effects of postexercise sera in BC cell viability is completely blunted, indicating the crucial role of catecholamines in inhibiting BC cells viability and tumor growth [124]. Their role in exercise-induced effects on BC cell viability has also been confirmed by MCF-7 and MDA-MB-231 treatment with different doses of EPI and NE, resulting in a dose-dependent growth inhibitory effect in both BC cell lines. Catecholamines have been shown to induce a dose-dependent phosphorylation of yes-associated protein (YAP) in MDA-MB-231 cells [125]; YAP is the main downstream target of the mammalian Hippo pathway and, when phosphorylated, it is retained in the cytoplasm. Hippo pathway is a tumor suppressor signaling cascade that regulates cell growth, and it has been shown to be a dysregulated pathway in several types of cancers, including BC, in which there is an activation of YAP oncoproteins and transcriptional coactivators with the PDZ-binding motif (TAZ) associated with tumor formation, growth and progression, metastasis, and drug resistance [126]. Dethlefsen et al. showed that the Hippo pathway is regulated by exercise-conditioned sera: incubation of BC cells with postexercise sera led to a time-dependent phosphorylation of YAP in MCF-7 BC cells and to a decreased expression of YAP target genes, due to phosphorylated-YAP cytoplasmic retention, in both MCF-7 BC cells and MDA-MB-231 TNBC cells [124]. Studies performed by Tumaneng et al. demonstrated that Hippo pathway is related to the mTOR signaling cascade: YAP mediates the effects of the Hippo pathway regulating target genes, including the miR-29; this miRNA family has been proven to inhibit PTEN, an upstream activator of mTOR [127]. In summary, the Hippo pathway can be activated by exercise through the production of the catecholamines EPI and NE and can inhibit BC cell growth through the action of YAP and miR-29, inactivating the mTOR pathway.

As mentioned above, several miRNAs have been found to be deregulated in TNBC cells and patients; evidence suggests that different types of exercise can regulate these miRNAs in different ways. One of these miRNAs is miR-21, which has been found to be upregulated in TNBC patients; it has an oncogene activity and plays a crucial role in tumor cell proliferation and invasion, repressing PTEN [128].

Nielsen et al. [129] showed how miR-21 level significantly decreased 3–5 days after endurance training (60 min of cycle ergometer exercise at 65% of *P*_max_, 5 times a week for 12 weeks), at rest. However, levels of miR-21 were also found to be upregulated immediately after a single exhausting cycling exercise at a low heart rate, just as it was after a training period of 90 days [130]. Discrepancies between data obtained by these two studies could be explained by the different types of exercise considered, as confirmed by Wardle et al. [131].

The microRNA precursor miR-146a has also been found to be an upregulated miRNA in TNBC tissues, and its level is related to tumor size and survival rate. Nielsen et al. [129] showed that miR-146a levels significantly decreased immediately after a single session of pedaling exercise performed at 65% of the maximal power output. In this case, depending on the different exercise considered, miR-146 levels can be dysregulated: after a single exhausting cycling exercise at a low heart rate, it has been found to be upregulated [130]. Variations in miR-146 levels when comparing strength or endurance exercise groups to controls were observed; levels increase in the endurance group, while they decrease in the strength group [131]. The downregulation of miR-146a after strength exercise was also confirmed by a study that involved a single strength exercise session performed at 70% of one-repetition maximum [132] in which the miR-146a level was found to have decreased 3 days after exercise.

In short, a subset of circulating miRNAs, including miR-21 and miR-146a, are associated with the whole-body adaptive response to differential forms of exercise and training. These miRNAs have been found to be upregulated in TNBC patients and related to the repression of PTEN or BRCA1 with consequent mTOR pathway activation. Hence, their downregulation with specific types of exercises could be a very promising approach to control TNBC initiation and progression.

## 4. Energy Intake in TNBC and mTOR Modulation

mTORC1 is a key regulator of cell growth and proliferation, and at the same time, it is also at the centre of nutrient regulation and utilization. In this regard, a large number of studies have demonstrated the role of excessive energy intake on cancer development, and by contrast, the protective effects of CR [133]. While the antitumorigenic effects of CR are well established, the mechanism behind this relationship is not completely clear, though it is believed that the tumor suppressive effects are mediated, as they are for exercise, by enhanced apoptosis, modulation of systemic signals such as IGF-1, insulin, metabolic, and inflammatory pathways, as well as by reduced angiogenesis [134]. Specifically, a large quantity of data points to the role of mTOR activation in cancer development through protein-induced IGF-1 signaling and to the beneficial effects of caloric and protein restriction not only on aging-associated diseases such as cancer but also on life span [135, 136] (Figure 1).

CR increases the level of the circulating adiponectin, which can exert anticancer effects through mechanisms that include an increase in insulin sensitivity, a decrease in insulin/IGF-1 and mTOR signaling via AMPK activation as well as a reduction in the proinflammatory cytokine expression via inhibition of the nuclear factor *κ*-light-chain-enhancer of activated B-cells (NF-*κ*B) [136, 137].

AMPK, as mentioned above, is an important mediator in the maintenance of cellular energy homeostasis, and recently, it has gained attention for its possible role as a metabolic tumor suppressor and in cancer prevention and control. Since AMPK phosphorylation is regulated by energy availability (AMP : ATP ratio), AMPK activators, such as metformin, CR, and aerobic exercise, reduce the incidence of cancer.

Leptin is a peptide hormone produced by white adipose tissue. It affects several tissues and acts on the hypothalamus to regulate appetite and energy expenditure. It also impacts carcinogenesis, angiogenesis, immune responses, cytokine production, and other biological processes [138, 139].

Intermittent CR is associated with the suppression of murine mammary tumor incidence and a decrease in the leptin-to-adiponectin ratio [139]. This ratio, when elevated, is related to metabolic syndrome and some cancers [140, 141]. In TNBC metastases, CR decreases proliferation, increases apoptosis, and downregulates the IGF1-1R pathway, coadiuvating canonical therapies [142]. Taken together, these findings show that dietary interventions can ameliorate the systemic milieu and tumor microenvironment. Chronic CR is not suitable for cancer patients at risk for weight loss, cachexia, and immunosuppression, but it can be substituted with intermittent CR, fasting-mimicking diets, low carbohydrate/ketogenic diets, or CR mimetic drugs.

Fasting and low carbohydrate diets have been shown to reduce side effects and to enhance the effectiveness of chemotherapy and radiation therapy in animal models, and there is a great deal of interest in the potential clinical value of these interventions.

Protein consumption has different effects on cancer mortality, which vary according to age, with an increased risk in middle age and a reduction in the elderly [143].

Protein restriction (PC) for the middle-aged followed by moderate protein intake in elderly subjects may increase longevity and health span since protein restriction is sufficient to reduce growth hormone receptor (GHR)-IGF1 activity and can reduce cancer incidence in model organisms regardless of energy intake [144].

Moreover, L-type amino acid transporter 1 (LAT1), which transports large quantities of neutral amino acids, was found highly expressed in human BC tissues. The upregulation of LAT1 plays an important role in BC progression because more amino acids are required for protein synthesis and cellular proliferation [145].

The activation of the mTOR/S6K1 signaling pathway depends on the availability of amino acids (AA), particularly branched chain AA, such as leucine, and also glucose [106]. Growth factor signals, which usually activate mTORC1 signaling, have little or no impact in the absence of AA.

Leucine deprivation causes an upregulation of insulin-like growth factor binding protein 1 through transcriptional activation and mRNA stabilization, probably decreasing the effects of IGF1 and thus lowering cell proliferation [146].

However, in most BC cell lines with constitutively activated Akt/mTOR signaling, leucine restriction is not efficient in inhibiting mTOR signaling since it is associated with activation of survival molecule Akt, making leucine deprivation an undesirable approach for BC therapy [146].

Glutamine is another AA involved in the regulation of the mTOR pathway inducing the uptake of leucine [147]. Tumor cells are more sensitive to amino acid deprivation than normal cells; thus, glutamine restriction and/or transporter inhibition decrease mTOR activity [147].

A novel therapeutic approach based on whey protein concentrate (WPC) supplementation for BC treatment has been suggested by Cheng et al. [148]. WPC is rich in bioavailable cysteine, which can be used for glutathione synthesis, and contains all nine essential AAs. WPC promotes muscle protein synthesis [149] and can be used as a nutritional supplement during chemotherapy [150]. WPC has also been shown to enhance rapamycin sensitivity in MDA-MB-231 TNBC cells, a cell line resistant to rapamycin and other mTOR inhibitors [148].

The combination of conventional therapies and *n-*3 polyunsaturated fatty acid (PUFA) supplementation (nutritional interventions) increases the sensitivity of tumor cells to conventional therapies, possibly improving their efficacy especially against cancers resistant to treatment, as suggested by D'Eliseo and Velotti [151]. Eicosapentaenoic acid (EPA) and docosahexaenoic acid (DHA) have anticancer effects on different cancer types by inducing apoptotic cell death in human cancer cells either alone or in combination with canonical therapies. EPA and/or DHA also have proapoptotic effects in both triple-negative [152] and ER+ BC subtypes [153], although when compared at the same dose, DHA appears to be more effective. This might be due to the structural differences between DHA and EPA. The proapoptotic effects occur with increases in plasma membrane incorporation and decreases in cell viability [152–154], PI3K/Akt pathway [155], and pEGFR activation [152].

In agreement, CR and other nutritional interventions could play an important role in support of conventional therapies to improve TNBC outcomes.

## 5. Exercise Prescription in BC Survivors

In general, reviews and meta-analyses tend to group PA and exercise interventions into general categories and rarely examine the specific exercise protocols employed in the studies. Therefore, which characteristics make an exercise protocol safe and effective for BC survivors and, particularly, for TNBC patients?

Since the 2009 roundtable consensus statement on exercise guidelines for cancer survivors [156], which outlined the situations in which deviations from the 2008 US Physical Activity Guidelines for Americans (PAGA) were appropriate and included relevant implementation strategies [157], exercise recommendations from several internationally recognized institutions, such as the American Cancer Society [158] and the National Comprehensive Cancer Network [159], have been published for BC survivors. Fortunately, all of the abovementioned publications have recently been reviewed within the framework for exercise prescription of the American College of Sports Medicine (ACSM) [160], along with others providing practical guidance for exercise prescription in these patients [161, 162]. ACSM's framework for exercise prescription employs the so-called FITT-VP principle [160], which considers the frequency (*F*), intensity (*I*), time (*T*), and type (*T*) of exercise and its volume (*V*) and progression (*P*) over time in an individualized exercise training program.

A detailed description of the FITT-VP principle for each type of exercise—i.e., aerobic, resistance, and flexibility—adapted to BCS needs is provided in Tables 1, 2, and 3. Note that the following guidelines should not be regarded as specific for BC patients because no studies, to date [163], have adopted (and/or reported) the proper application of the principles of specificity, progression, overload, initial values, and adherence, within their exercise interventions. Therefore, although specific exercise guidelines for cancer survivors still need to be outlined, particularly for TNBC survivors, the following information represents the most up-to-date adaptations of the PAGA to BCS, including TBNC patients. Improving the reporting of exercise prescriptions will also allow for more specific recommendations regarding types and doses of exercise for BCS (and, hopefully, for the TNBC subgroup), in order to identify effective exercise interventions to be delivered to this growing community.

## 6. Benefits of Exercise Pre- and Postdiagnoses

Humans have not been “designed” for a sedentary lifestyle. The absence of an adequate level of PA puts us at increased risk of developing cancer. This has been highlighted by the European Breast Cancer Conference [164], issued an important statement: regular PA reduces the risk of BC for woman of any age and body weight by 12%.

PA as a nonpharmacological treatment to combat the collateral effects associated with BC is under considerable scientific attention [160, 165, 166]. To allow physicians to prescribe PA to patients before and after treatment, scientific clarity and evidence supporting the thesis that PA programs reduce the damaging effects of cancer and its treatment are needed. Very little is known about the effect of exercise on TNBC outcomes, but data suggest that pre- and postdiagnosis PA may be one of the factors, which, if appropriately prescribed, could bring benefits to patients.

Generally, TNBC has poor treatment outcomes because of a lack of receptor targets for conventional drugs to act upon. However, there is irrefutable evidence of the effectiveness of regular PA in primary and secondary prevention of premature death from any cause, including BC. Thus, different types of exercise can influence the prevention and progression of disease through several common mechanisms such as reduction of insulin resistance and improvement in immunity and cardiovascular function. Research in humans shows that exercise can regulate inflammation [13, 167], oxidation [168, 169], and gene expression [170].

Together with the potential mechanisms underlying the effects of exercise on breast carcinogenesis, Thompson [12] proposed three interesting hypotheses: (i) the hormesis hypothesis: oncological response to exercise is antithetical to a physiological cellular stress response; (ii) the metabolic reprogramming hypothesis: exercise reduces the glucose and glutamine available to mammary carcinomas, inducing apoptosis and reversing tumor-associated metabolic program; and (iii) the mTOR network hypothesis: exercise inhibits carcinogenesis by suppressing the activation of the mTOR signaling network in mammary carcinomas.

Recent investigations have revealed that the most active women had, on average, a 25–30% lower BC risk than women in the lowest category of recreational PA [171]. Data from the California Teachers Study (CTS) suggest that PA has a protective role in prediagnoses and may reduce a woman's risk of BC, especially the TNBC subtype. An analysis of the risk index (HR) associated with variations in the amount of PA hours among TNBC women yielded significant results. The HR results show significant associations when moderate-intense activity is considered as the only variable. When they are considered as separate variables, there are no statistically significant associations between moderate activity and TNBC, whereas intense activity is inversely associated with TNBC [172]. The reduction risk associated with baseline strenuous recreational PA was statistically significant among overweight or obese pre- or postmenopausal women, but not among their leaner counterparts.

In patients with BC postdiagnosis, acute and chronic symptoms, such as muscle mass loss, fatigue, weight gain, hormone alterations, bone loss, cachexia, and adverse psychological effects, may all be favourably influenced by regular exercise. A prospective cohort study analysed modifiable lifestyle factors, including exercise, associated with total mortality and recurrence/disease-specific mortality in patients with TNBC [173]. The association between TNBC prognoses and exercise postdiagnosis yielded important results: women who engaged in exercise regularly during the first 6 months postdiagnosis had a lower risk of total mortality and recurrence/disease-specific mortality, with adjusted HRs of 0.58 and 0.54, respectively. In addition, those who engaged in PA for a long time (2.5 h/wk) or women who exercised ≥7.6 metabolic equivalent hours/wk had a reduced risk of all causes and recurrence/disease-specific mortality compared with nonexercisers. Survivors who maintain a healthy weight and stay physically active have a better response to treatment and better survival outcomes. Thus, it is necessary to identify an appropriate promotion and prescription of regular PA for BC survivors in order to improve their prognosis, response to therapy, and quality of life. As previously described, the mTOR signaling pathway is differentially regulated by different exercise modalities, and it represents one of the main key regulators of the protective effects of exercise.

## 7. Conclusions

In this review, we presented new insights into the downregulation of mTOR signaling in TNBC by exercise and CR. It has been shown that mTOR network inhibition is mostly mediated through the effects of CR and vigorous PA as well as long-term exercise, which decrease the level of circulating growth factors and hormones.

During exercise, the body is exposed to different types of stressors, including temperature, metabolism, hypoxic, oxidative, and mechanical stress. These stressors initiate biochemical targets, which in turn actuate different signaling pathways that regulate gene expression and adaptive responses. Beneficial adaptation likely depends on the basal state of oxidative stress and inflammation at the beginning of exercise training. In turn, this basal state may depend on the periodization of training and recovery, together with age, health status, and diet.

Exercise, as a hormetic agent, has the potential for beneficial energy upregulation. The dose response effects are complex and reflect activation of major defensive pathways in both systemic and local environments. A mitohormetic stimulus that occurs through a physiological cellular stress adaptation and AMPK activation across hormetic control circuits, such as increase of oxidative metabolism, mitochondrial biogenesis, angiogenesis, immune regulation and a decrease in BMI, and insulin secretion, are induced by exercise. Moreover, PA increases glucagon, catecholamines, and other hormones and influences miRNAs involved in cancer. Exercise as well as CR limit glycaemia and glutamine availability to mammary carcinomas, inducing apoptosis and reversing malignancy-associated metabolic programming. It is also known that intratumoral metabolism is regulated by exercise, but how this affects tumor growth and metastatic rate is not clearly understood. Although the signal for these hormonal and autonomic changes has been partially described in ex vivo experiments, such changes are difficult to transfer in vivo. Currently, there is an agreement in the literature that there is a role for exercise as a coadiuvant “medicine” in canonical therapies and that it has an increasingly protective tumorigenic effect. In this context, PA needs to be broken down into its main components: frequency, intensity, time, and type; however, the dose-dependent effects of each of these components on cancer protection via mTOR inhibition are still unclear. Most data suggest that both vigorous and long-term PA in adulthood may reduce a woman's risk of mammalian cancer, especially the TNBC subtype.

Finally, we can assert that there is a sufficient evidence showing that sedentary behaviour and nutritional risk factors for TNBC are modifiable. Hence, the suggestions regarding the modification of such risk factors highlighted in this review could have wide-ranging implications for society and may improve public healthcare cancer management. Accordingly, we would like to emphasize the importance of promoting physically active lifestyles to reduce the risk of relapse in TNBC. Fostering active lifestyles can provide important support during conventional cancer treatment, preventing the potential negative impacts on patients' physical condition, as well as their emotional and social well-being.

## Figures and Tables

**Table 1:** Aerobic (cardiorespiratory endurance) exercise recommendations.

Intensity (*I*)	Frequency (*F*)	Time (*T*) (duration)	Type (*T*) (mode) (examples)	Volume (*V*) (quantity)	Progression (*P*) (rate of)	Specific notes
Light: 30–39% VO_2_R/HRR; 57–63% HR_max_; 9–11 RPE.	At least 5 d wk^−1^.	30 to 60 min each session (i.e., at least 150 min wk^−1^).	Continuous and rhythmic exercises that involve major muscle groups (walking, cycling, slow dancing, jogging, running, rowing, stepping, fast dancing, etc.).	≥500–1,000 MET min wk^−1^.	Increase gradually any of the FITT components as tolerated by the patient (gradual progression is required to minimize the risks of muscular soreness, injury, undue fatigue, and the long-term risk of overtraining). Initiate increasing exercise duration (as tolerated): an example for healthy people is adding 5–10 min every 1–2 wk over the first 4–6 wk and adjusting upward over the next 4–8 months to meet the recommended FITT components, but slower progression may be needed for BCS.	If tolerated without adverse effects of symptoms or side effects, moderate to vigorous intensity and 3–5 d wk^−1^ frequency are recommended, but lower (light) intensities and frequencies are still beneficial when the current physical activity level is low. Avoid prescribing and monitoring intensity using %HRR (using %HR_max_ or RPE is recommended in BCS). Be aware of fracture risk, because bone is a common site of metastases in breast cancer: BCS with metastatic disease to the bone will require modification of their exercise program (e.g., reduced impact, intensity, and volume) given the increased risk of bone fragility and fractures.
Moderate: 40–59% VO_2_R/HRR; 64–75% HR_max_; 12-13 RPE.	At least 5 d wk^−1^.	30 to 60 min each session (i.e., at least 150 min wk^−1^).				
Vigorous: 60–89% VO_2_R/HRR; 76–95% HR_max_; 14–17 RPE.	At least 3 d wk^−1^.	20 to 60 min each session (i.e., at least 75 min wk^−1^).				

Modified from [160]. VO2R: oxygen uptake reserve, calculated as the difference between maximal oxygen uptake and resting oxygen uptake; HRR: heart rate reserve, calculated as the difference between maximal heart rate and resting heart rate; HR_max_: maximal heart rate; RPE: rate of perceived exertion on the 6–20 scale; MET-min: metabolic equivalents (MET) of energy expenditure for a physical activity performed for a given number of minutes (min), calculated as MET × min; FITT: frequency, intensity, time, and type of exercise.

**Table 2:** Resistance (strength) exercise recommendations.

Intensity (*I*)	Frequency (*F*)	Time (*T*) (duration)	Type (*T*) (mode) (examples)	Volume (*V*) (quantity)	Progression (*P*) (rate of)	Specific notes
Light: 30–49% 1-RM.	2-3 d wk^−1^.	Depends on exercise volume (number of sets, repetitions for each set, and rest intervals in-between) and is not associated with effectiveness.	Any form of movement designed to improve muscular fitness by exercising a muscle or a muscle group against external resistance: exercise and breathing techniques are of paramount importance and symptom-limited ROMs should be adopted according to BCS responses to exercise (free weights, resistance machines, weight-bearing functional tasks, etc.).	2–4 sets of 8–15 repetitions (at least 1 set of 8–12 repetitions can be effective in BCS) with 2-3 min rest between sets.	BCS should start with a supervised program of at least 16 sessions and very low resistance (<30% 1-RM), and progress with smallest increment possible (e.g., 2–10% 1-RM, depending on muscular size and involvement, is recommended for healthy adults). If a break is taken, lower the level of resistance by 2 wk worth for every week of no exercise.	No upper limit on the account of weight to which BCS can progress. Individuals with lymphedema should wear a compression sleeve during resistance training activity. Watch for arm/shoulder symptoms including lymphedema and reduce resistance or stop specific exercises according to symptom response. Be aware of risk of fracture (see aerobic exercise for details).
Moderate: 50–69% 1-RM.	2-3 d wk^−1^.					
Vigorous: 70–84% 1-RM.	2-3 d wk^−1^.					

Modified from [160]. 1-RM: one-repetition maximum, i.e., the load that can be lifted one time only; ROM: range of motion; BCS: breast cancer survivors.

**Table 3:** Flexibility (stretching) exercise recommendations.

Intensity (*I*)	Frequency (*F*)	Time (*T*) (duration)	Type (*T*) (mode) (examples)	Volume (*V*) (quantity)	Progression (*P*) (rate of)	Specific notes
Stretch to the point of feeling tightness or slight discomfort.	≥2-3 d wk^−1^ (stretching on a daily basis is most effective).	Hold a static stretch for at least 10–30 s (30–60 s may confer greater benefit). Accumulate a total of 60 s of stretching for each flexibility exercise by adjusting time/duration and repetitions (see volume) according to individual needs.	Stretching exercise that increases the ability to move a joint through its complete ROM (provided individual specific conditions are accounted for) (static active flexibility, static passive flexibility, dynamic flexibility, ballistic flexibility, proprioceptive neuromuscular facilitation, etc.).	Repeat each exercise 2–4 times in order to attain the goal of 60 s stretch time (e.g., two 30 s stretches or four 15 s stretches). A stretching routine can be completed approximately in ≤10 min.	Optimal progression is still unknown.	BCS should focus on joints in which a loss of ROM occurred because of surgery, corticosteroid use, and/or radiation therapy. Flexibility exercises are most effective when the muscles are warm.

Modified from [160]. ROM: range of motion; BCS: breast cancer survivors.
